# Assessing effectiveness of serious game training designed to assist in upper limb prosthesis rehabilitation

**DOI:** 10.3389/fresc.2024.1353077

**Published:** 2024-01-29

**Authors:** Bart Maas, Corry K. Van Der Sluis, Raoul M. Bongers

**Affiliations:** ^1^ Department of Human Movement Sciences, University Medical Center Groningen, University of Groningen, Groningen, Netherlands; ^2^ Department of Rehabilitation Medicine, University Medical Center Groningen, University of Groningen, Groningen, Netherlands

**Keywords:** prosthesis, rehabilitation, serious games, upper limb prosthesis, task specificity

## Abstract

**Introduction:**

Controlling a myoelectric upper limb prosthesis is difficult, therefore training is required. Since training with serious games showed promising results, the current paper focuses on game design and its effectivity for transfer between in-game skill to actual prosthesis use for proportional control of hand opening and control of switching between grips. We also examined training duration and individual differences.

**Method:**

Thirty-six participants were randomly assigned to one of three groups: a task-specific serious game training group, a non-task-specific serious game training group and a control group. Each group performed a pre-test, mid-test and a post-test with five training sessions between each test moment. Test sessions assessed proportional control using the Cylinder test, a test designed to measure scaling of hand aperture during grabbing actions, and the combined use of proportional and switch control using the Clothespin Relocation Test, part of the Southampton Hand Assessment Procedure and Tray Test. Switch control was assessed during training by measuring amplitude difference and phasing of co-contraction triggers.

**Results:**

Differences between groups over test sessions were observed for proportional control tasks, however there was lack of structure in these findings. Maximum aperture changed with test moment and some participants adjusted maximum aperture for smaller objects. For proportional and switch control tasks no differences between groups were observed. The effect of test moment suggests a testing effect. For learning switch control, an overall improvement across groups was found in phasing of the co-contraction peaks. Importantly, individual differences were found in all analyses.

**Conclusion:**

As improvements over test sessions were found, but no relevant differences between groups were revealed, we conclude that transfer effects from game training to actual prosthesis use did not take place. Task specificity nor training duration had effects on outcomes. Our results imply testing effects instead of transfer effects, in which individual differences played a significant role. How transfer from serious game training in upper limb prosthesis use can be enhanced, needs further attention.

## Introduction

The ability to perform goal directed movements in daily life of individuals with upper limb absence is lower compared to an able-bodied person (1). The use of a state-of-the-art prosthesis should be able to restore at least part of the functionality, but rejection rates have not been reduced in the last two decades (2–7), despite substantial technological developments. A possible reason that prostheses do not provide functionality to an acceptable level might be due to low levels of prosthesis control, which in turn might result in rejection (2, 8). This conjecture is in line with the idea that using an upper limb prosthesis to perform a task is difficult because the prosthesis is controlled in a different way than a natural hand. Control can be practiced using serious game training, although in the current literature there is still a debate on what training is most effective (9–20). The current paper focuses on what type of serious game training could improve prosthesis control most effectively.

Current state-of-the-art prostheses consist of a bionic hand with multiple grip possibilities. Such a type of bionic hand is controlled by electromyography (EMG) signals produced by activity of the remnant muscles in the stump (i.e., a myoelectric upper limb prosthesis). The electric current that is produced by activating muscles can be used to operate the motors in the prosthesis hand and is called a myosignal. In most current-day applications of myoelectric upper limb prostheses muscle activity is measured by two electrodes attached to the skin above the flexors and extensors. To control the multi-grip hand of a myoelectric prosthesis in a most dexterous way two types of control are needed, proportional and switch control (21). Proportional control means that the amount of muscle activation is proportional to the speed with which the prosthetic hand opens and closes. Switch control means that when the user provides a correct trigger, for example a co-contraction (when the wrist flexor and extensor are activated at the same time following a simultaneous activation signal), the prosthetic hand switches from one grip type to another (for example from the tripod grip to the lateral grip). Most users of multi-grip prosthesis hands need to learn to master both proportional and switch control to use their bionic hand to its full potential; a skill that can be trained as shown by previous research (22–29). However, producing adequate myosignals is not an easy skill to learn (13, 14). Using serious games as training could provide interesting opportunities due to benefits such as more autonomy for users, more engagement, and less need for assistance of a therapist compared to conventional training programs.

### Training

Serious games are most often computer games that can be used for education or training, with entertainment being a secondary purpose (30, 31). Benefits of using serious games for training are increased motivation compared to conventional training, inclusion of individualized and augmented feedback and a relative independence in executing the rehabilitation program (32, 33). Furthermore, serious games allow for training of the control of the prosthesis before the prosthesis is available. This enables users to start training early since it might take weeks or months before a prosthesis becomes available. Such training might have benefits since it could exploit neural plasticity processes at work after an amputation (Di Pino et al., 2009; Malone et al., 1984.). Hence, serious games seem to provide ample advantages over conventional training of prosthesis use, especially in the pre-prosthetic phase.

The serious games used in the domain of upper limb prostheses use myosignals to control the avatar in the game in a similar manner as these signals are used to control an actual prosthesis (11, 12, 33, 34–36). Therefore the assumption is that when one improves their myocontrol in the game, the learned skill will transfer to actual prosthesis use and as a consequence users will improve their ability to control the prosthesis. Even though several studies have been performed on serious game training for upper limb prosthesis in the past decade (11, 12, 16, 17, 19, 20, 29, 34, 37–39), the most effective game design to facilitate transfer from the game to actual prosthesis use has not been established. One reason for this might be that people differ in the way their individual motor learning processes are stimulated best as well as in their overall motor learning capacities (40–45). An important advantage of serious games is that in-game performance immediately affects the feedback provided to the user, hence these games are inherently individualized. Moreover, the different levels that can be presented allow for opportunities to tailor training conditions (e.g., variations in order of training tasks or type of feedback) to each individual to optimize transfer of training to actual prosthesis use. How the design of serious games affects transfer is currently unknown, hence, the current paper takes inspiration from motor learning principles to design games that may optimize transfer.

### Motor learning: perception-action coupling

Previous research has shown that individuals differ in the performance on different myocontrol tasks (14). These results indicate that performance is specific for the relation between perception (i.e., the perceptual information exploited to perform a specific task) and action (i.e., the movements or muscle activations to complete a task) (46, 47). This means that when a new task has to be learned, a new coupling between perception and action has to be learned. Transfer of skill will only occur when the same perception-action coupling is present in both the training and the actual task.

A task-specific serious game for prosthesis control should resemble tasks that a prosthesis user might encounter in their day-to-day life, such as opening and closing of a prosthesis hand (i.e., proportional control) or switching between different grips (i.e., switch control) (10, 17, 21). Transfer effects were found previously after training with a task-specific serious game, but not after training with a non-task-specific serious game (9).

Until now, task-specificity in prosthesis use has only been investigated for proportional control. It is questionable what task-specific training means for switch control, in particular since Heerschop et al. has shown that different perception-action couplings seem to underly proportional control and switch control (14). Moreover, in a setting where both proportional and switch control were required, Tabor et al., 2018 suggested that transfer of skill does not depend on task-specificity but on the duration of the training period. Tabor et al. indicated that the short duration of the training in Van Dijk et al. was responsible for not finding transfer effects after practicing with the non-task-specific serious game. However, Tabor et al. did not test the transfer of training effects in actual prosthesis use since no pre-test post-test design was used and their analyses were restricted to the change in the myosignals. Hence, an experimental design in which task-specificity, duration of training, functional testing with an actual prosthesis and analyses of the myosignals are combined would enable us to make more decisive conclusions.

### Research questions

Does the transfer from myocontrol training in a serious game to actual prosthesis use differ among three training groups (task-specific serious game, non-task-specific serious game, and control group), does the transfer differ between shorter and longer training programs, and do these factors interact? These research questions were asked for tasks in which only proportional control was required as well as for tasks in which both proportional and switch control were used. For the tasks in which both proportional and switch control were required it was also examined whether individual participants differed in learning.

Furthermore, we investigated whether during training the features of the myosignal, used to produce a switch, differed between both serious gaming groups, between the different training durations and how these factors interacted.

## Methods

### Ethical approval

The study was approved by the Local Ethical Committee of the department of Human Movement Sciences of the University Medical Center Groningen, Groningen, the Netherlands (local Research Registry number: 201900815). All participants received an information letter prior to the pre-test and were asked to sign an informed consent before the start of the experiment.

### Participants

Participants were recruited from the student population of the University Medical Center Groningen and University of Groningen. Eligibility criteria were that they were able-bodied, right-handed, had corrected to normal vision, were free of any disorders to their arm or upper body and had no prior experience with a myoelectric prosthesis simulator. Handedness was tested using the Edinburgh Handedness Inventory (48).

### Design

The experiment was conducted in 13 sessions which consisted of three test sessions (pre-test, mid-test and post-test) and ten training sessions, with five training sessions between each test session. Sessions were conducted on separate days with a minimum of three and a maximum of five sessions per week. Participants were randomly assigned using a random number generator to one of three groups: the Task-Specific (TS) group, the Non-Task-Specific (NTS) group and the Control (C) group. Participants all performed the same tests in the test sessions but the training differed based on their group.

A power analysis was performed with G*Power using the data from (9) to determine the number of participants needed. The analysis showed that to reach a power of 0.85 and an alpha of 0.05 the number of participants needed was 33.

### Materials and procedures

For the pre-test, mid-test and post-test, participants used a myoelectric prosthesis simulator in order to closely resemble a below-elbow myoelectric prosthesis. The simulator consisted of an iLimb Ultra Revolution hand (2013, Touch Bionics, Össur) that was placed distal to the user's hand and was attached to a splint with an open cast wherein the participants' forearm could be placed. The splint was adjustable in length and the cast was attached to the participants' forearm using a leather sleeve which was closed using Velcro straps (26, 27). Two electrodes (13E200 electrodes, MyoBock, Otto Bock Healthcare products, Austria) were embedded in the Velcro leather sleeve around the arm. The electrodes were placed on the most prominent muscle bellies of the wrist flexors and extensors during contractions, which were found using palpation after instructing each participant to move their wrist. The location was marked with a waterproof pen in the pre-test and was repeated over the training sessions if the marking faded. Participants also took a waterproof pen home to be able to keep the marking visible. The electrodes measured muscle activation so that the prosthetic hand could be closed and opened by activating the wrist flexors and extensors respectively, which is similar to the control of an actual upper-limb prosthesis. In addition, by contracting the flexor and extensor at the same time in a short burst, i.e., a co-contraction, the prosthetic hand could change the grip types. The requirements for a correct co-contraction were that both contraction peaks of the wrist flexors and extensors needed to reach the threshold of 40% of maximum voluntary contraction and within 350 ms of each other.

To assess prosthesis control in the pre-test, mid-test and post-test the following tests were used: the Cylinder test (49), part of the activities of daily living (ADL) section of the Southampton Hand Assessment Procedure (SHAP (50);, the Clothespin Relocation Test (CRT (51); and the Tray test (52).

#### Cylinder test

The Cylinder test was the same test used by (9, 49). For this test, participants had to grasp three different wooden cylinders with the prosthesis simulator in five trials (order was block randomized), while the angle of the hand opening was measured by a goniometer (sampling rate 2000 Hz, Cermet PC300 potentiometer, Contelec, Switzerland). Two legs were attached to the goniometer, one to the housing and one to the slider. The leg of the housing was attached to the thumb of the prosthesis hand and the leg of the slider was attached to the index finger. The potentiometer was connected to an NI-USB 6009 data acquisition device (National Instruments Corporation, USA). The angle measured by the potentiometer was sent to a laptop. The wooden cylinders were 10 cm in height and had a diameter of either 2 cm, 4 cm or 6 cm. Before the test started, participants had to maximally open and close the prosthesis hand to calibrate the signals of the goniometer to the maximal and minimal aperture. Participants had to start with a closed prosthesis hand resting on a pressure sensor located to the right of the cylinder and at the closer edge of the table. They were then asked to grasp a wooden cylinder, which was placed in front of them at 21 cm from the edge of the table, lift a grasped cylinder about 5 cm, and place it back down. The cylinder was constantly within the participants' sight and the movement to grasp the cylinder was made parallel to the frontal plane. Therefore participants did not have to open the hand in order to see the cylinder, as sometimes is necessary in daily life when grasping objects. Participants were instructed to be as accurate as possible while grasping the cylinders and not focus on speed. The maximum opening angle of the hand was measured during the grasping of each cylinder.

#### SHAP

Two tasks from the ADL section of the SHAP were used: page turning and key turning. These tasks were chosen to push participants to learn difficult tasks in which they could improve their performance, although in daily life the prosthesis is not often used for these tasks. An important reason to use these tasks is that they enabled to test both proportional and switch control at the same time. For each task the prosthesis hand was initially placed in a tripod grip and it was at the participant's choice to switch to a lateral grip to complete the task. The tasks were to use the prosthesis hand to flip over a paper and to turn over a key in a lock, respectively. The completion time of each separate task was recorded by the participant by pressing the button on the timer (which was provided in the SHAP test) with the hand of the prosthesis simulator to start the task, and pressing the button again after they completed the task.

#### CRT

For the CRT the standard equipment was used which was a set-up with a vertical and a horizontal rod with six red clothespins, three on each rod. Participants started in a tri-pod grip and were asked to grab a clothespin from the vertical rod and place it back on the same rod. Subsequently, participants needed to switch to a lateral grip and were asked to grab a clothespin from the horizontal rod and place it back on the same rod. Then participants had to switch back to the tri-pod grip and grab the second clothespin on the vertical rod. This process was repeated until all six clothespins were grabbed and placed back. The total completion time of all six clothespins was recorded with a stopwatch by the researcher.

#### Tray test (52)

**Figure 1:**
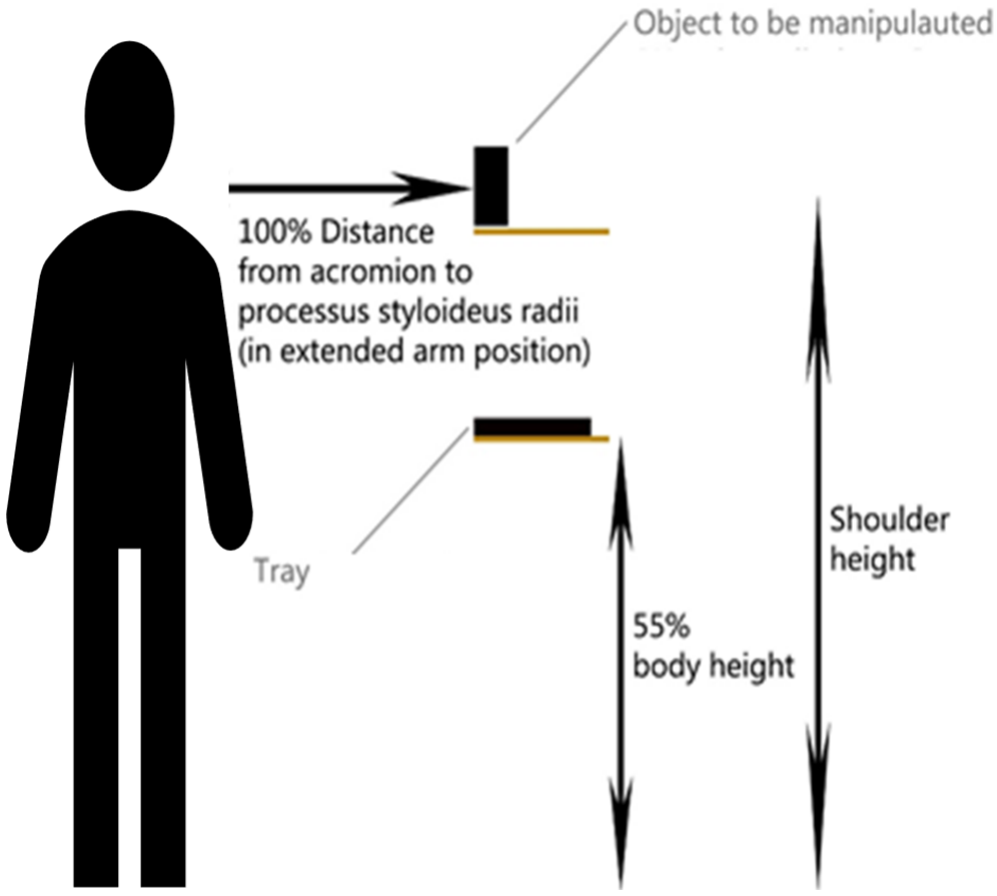
Experimental set-up for the tray test. The cylinder (i.e. object to be manipulated) was put on a shelf at the height of the shoulder and the tray was put on a shelf at the height of 55% percent body height.

The set-up for the Tray test consisted of two shelves that were adjustable in height, a tray and a wooden cylinder (10 cm in height, 4 cm in diameter). Before the Tray test started, the top shelf was placed at the participant's shoulder height and the bottom shelf was placed at their waist height (55% body height). Then the cylinder (i.e., “object to be manipulated”, see Figure 1) was placed on the top shelf and the tray on the bottom shelf. Participants started in a tri-pod grip and were asked to use the prosthesis hand to grab the cylinder and place it on the tray, then switch to a lateral grip and use both their prosthesis hand and intact hand to place the tray on the top shelf. The completion time of one single trial was recorded using the timer from the SHAP test. The Tray test was developed by Franzke et al. (52) and added to include a bimanual task, since it is known that most prosthesis users use their device mainly for bimanual tasks (4, 53).

In between test sessions, participants performed ten (two times five) training sessions using a serious game. The TS group and the NTS group trained myocontrol without the use of the prosthesis simulator, but in the same way as an actual prosthesis is controlled. The C group used a computer mouse and keyboard to play the games. Muscle activity was measured using electrodes (13E200 electrodes, MyoBock, Otto Bock Healthcare products, Austria) that were placed, after cleaning and dampening with an alcohol swab, on the location that was marked in the pretest and were held in place using a sweatband. The electrodes had a gain setting which was set at 4 initially and was adjusted if necessary for each participant individually at the start of the training session. The electrodes were connected to a MyRio (National Instruments, USA), on which custom-built LabView soft-ware (National Instruments Corporation, USA) digitally filtered the signals (band filter, cut-off frequency 10 Hz; low-pass filter, cut-off frequency 35 Hz). After the electrodes were placed, the myosignal needed to be calibrated which was done by determining the amplitude of the myosignal maximum voluntary contraction (MVC). The myosignal was then scaled so that the minimum movement speed in the games was 10% of the MVC and the maximum movement speed was 75% of MVC (9).

#### The task-specific group

**Figure 2:**
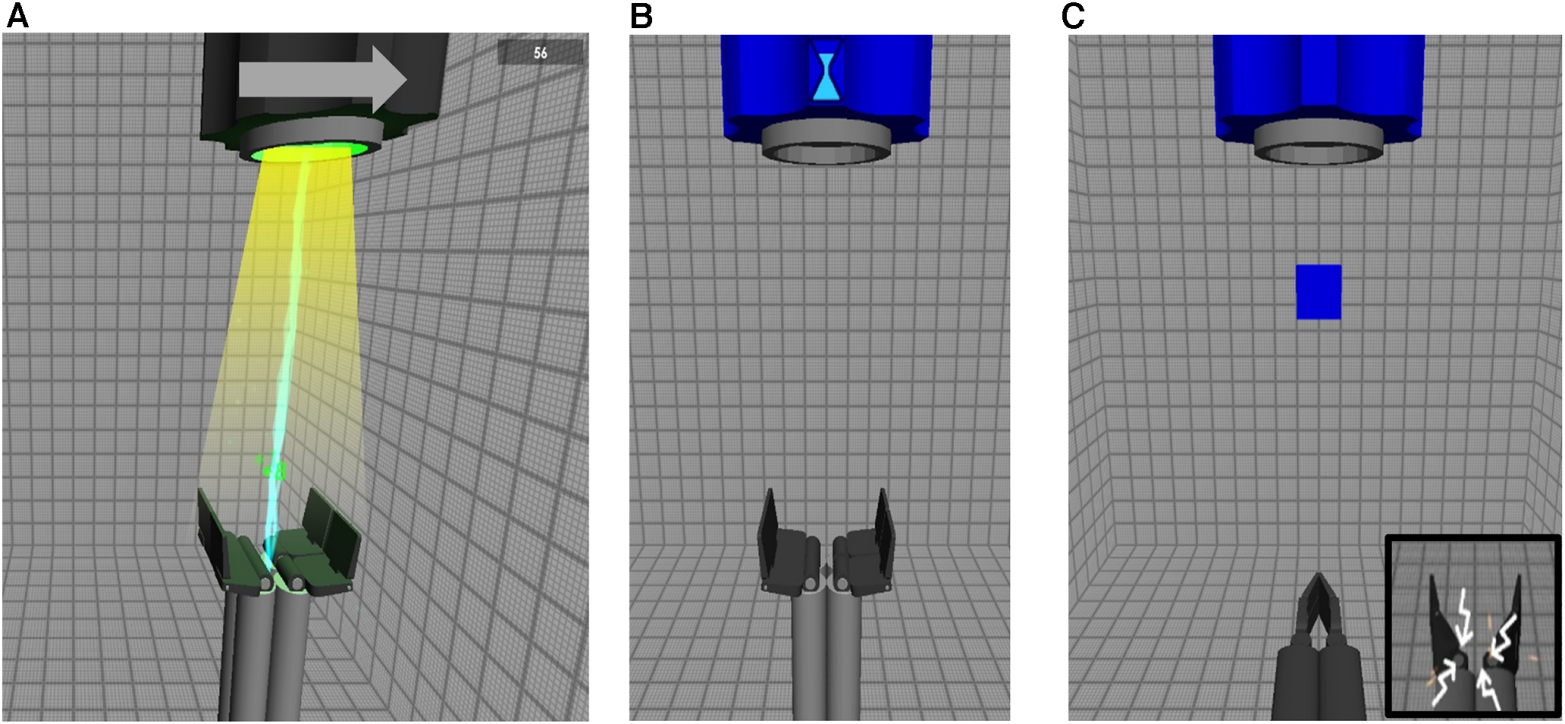
All three parts of the task-specific game. (**A**) The arrow at the top indicated whether the barrel at the top would move to the left or to the right. The platform, at the bottom, is controlled by the participant using myosignals and the participant is instructed to follow the light beam with the correct speed which made the lightning appear from the barrel. When the lightning appeared, the participants were scoring points shown in green overlapping the lightning. (**B**) An hourglass is shown at the top of the screen which is slowly depleting. This represented the amount of time the participants had to make a correct co-contraction. (**C**) After a correct co-contraction is made, the platform changes into a grabber that can open and close using the same myosignals. A block is dropped from the barrel at the top of the screen and the participants were instructed to catch the block by opening and closing the grabber. If the grabber would open too far (i.e. 1.7 times the width of the block) the grabber would shoot out sparks (bottom right).

The game used in the training sessions of the Task-Specific group consisted of three parts with an overall objective to control an avatar, either a platform or a grabber, see Figure 2. The participant could move or open and close the avatar by using proportional control, meaning that the speed of the avatar was proportional to the amplitude of the myosignal. The avatar could switch from the platform into the grabber by using switch control (i.e., a co-contraction).

In the first part (see Figure 2A), the objective was to move the platform horizontally to follow a beam of light coming from a barrel located at the top of the screen. The direction in which the light beam would move was indicated by an arrow pointing either to the right or the left. The platform could move to the left or the right by activating the flexors or extensors of the wrist, respectively. The goal was to move the platform at such a speed that it stayed within the light beam until it reached the edge of the screen. When this was done correctly, lightning would appear and points would be scored, as can be seen in the figure.

In the second part (Figure 2B), the objective was to make a co-contraction in a set amount of time indicated by the hour glass at the top of the screen. If a correct co-contraction was made within the time limit, the platform changed into a grabber. If an incorrect co-contraction was made, the grabber would “break” which meant that the third part of the game could not be played. If this happened, the game would continue and show the third part with a block falling from the barrel, but the participant would be unable to open or close the grabber because it was “broken”.

In the third part (Figure 2C), the objective was to use the grabber to catch a falling block from the top of the screen. The grabber could be opened and closed by activating the extensors and flexors of the wrist using proportional control. The participant needed to adapt the aperture of the grabber to the width of the blocks. If the grabber was opened 1.7 times the width of the block, the grabber would vibrate and shoot sparks (see bottom right of Figure 2C), as an indication that the hand opened too far. If the grabber opened 1.9 times the width of the block, the grabber would “break” and stop working. This particular feature was added to the game to guide participants to scale the aperture of their prosthesis hand to the object that should be grasped since this ability is an indication of better prosthesis control (see Bouwsema, Sluis, et al., 2010; Van Dijk et al., 2016b. The diameter of the falling blocks came in three sizes which were presented in random order. The blocks also varied in fragility, which was indicated by the number of cracks on the block. Blocks with more cracks would break more easily if they were grabbed with too much force. This required the user to vary in the closing speed of the grabber, since a higher closing speed implied a higher grasping force. After the third part the game would restart from the beginning.

Participants trained for twenty minutes in each training session. Three levels of the game were created to increase motivation of the participants. The levels increased in difficulty by moving the beam of light faster, dropping the blocks faster and making them more fragile. Participants trained in the first five training sessions for ten minutes with level one, then ten minutes with level two. In the last five training sessions they trained for ten minutes with level two and for ten minutes with level three.

#### Non-task-specific group

**Figure 3:**
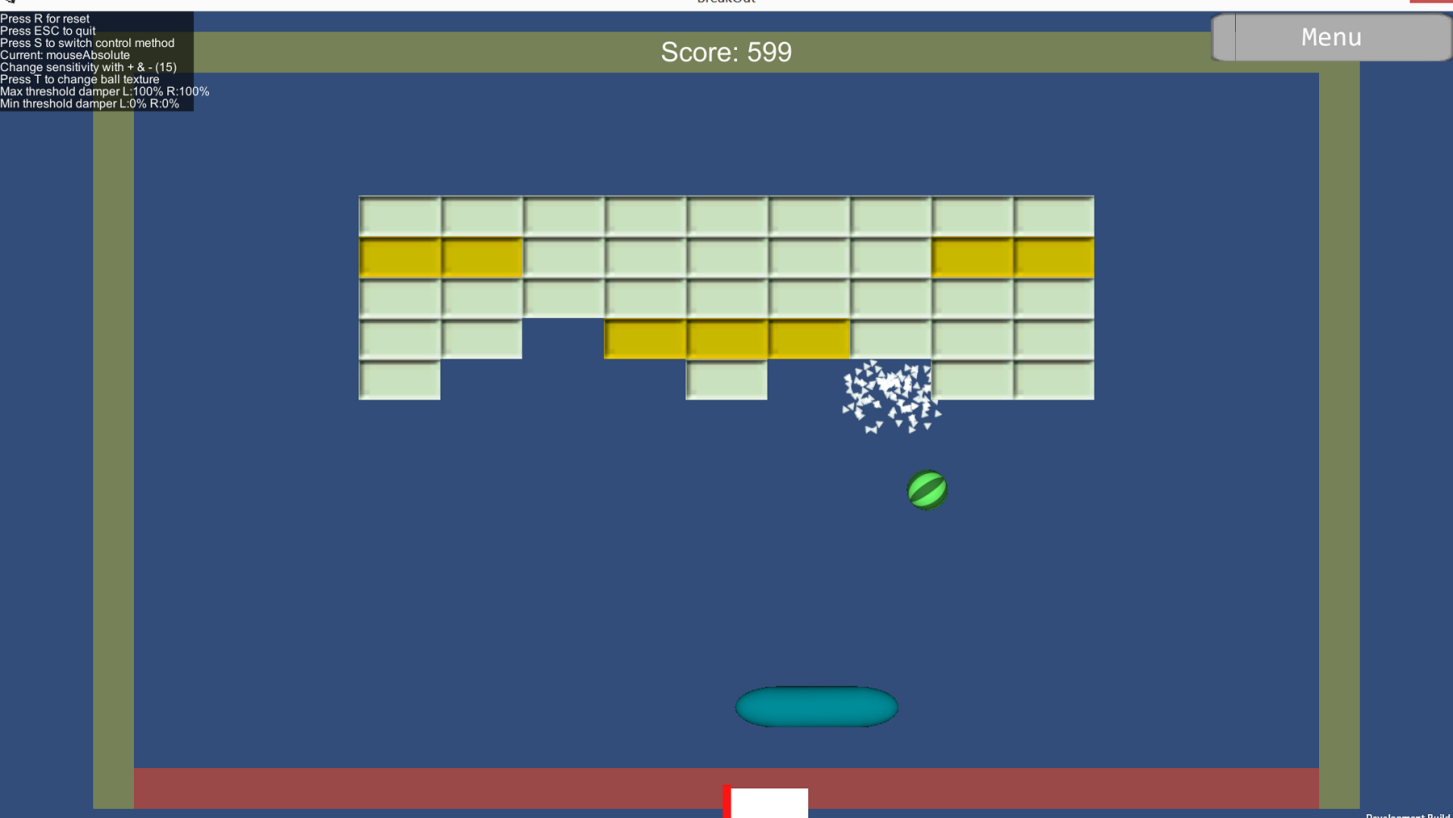
The breakout game. The paddle at the bottom was controlled by myosignals of the flexor (for moving left) and the extensor (for moving right) of the wrist. The paddle was used to break the blocks at the top. Some blocks were stronger and needed to be hit twice, indicated by a darker color. There were three levels with each level increasing in number of blocks and stronger blocks.

The game used in the Non-Task-Specific group was the Breakout game (Figure 3) as was used by Van Dijk et al., 2016b. The objective of the game was to keep the ball from hitting the ground by moving the paddle horizontally while at the same time hitting the blocks at the top of the screen. The paddle could be moved left and right by activating the flexors and extensors of the wrist, respectively, using proportional control. Subsequently, participants trained making co-contractions for three minutes. This was done by asking participants to look at the computer screen where their myosignal was shown and making as many co-contractions as possible. A correct co-contraction was indicated on the screen. Feedback on how to improve was provided by the researcher if necessary.

Participants trained for seventeen minutes with the Breakout game. The game consisted of three levels where the number of blocks increased with each level. During a training session, participants would complete all levels in ascending order and then start again at level one. In the seventeen minutes participants played each level two or three times.

#### Control group

Participants in the control group played both games from the TS and the NTS groups for 10 min each, but used a computer mouse and keyboard instead of muscle activation to control the avatar.

### Data analyses

All outcome measures presented in this section were computed with customized scripts using Matlab (2020a, The Mathworks Inc., USA). The Cylinder test was analyzed in a similar manner as Van Dijk et al. 2016. To this end, the maximum opening angle of the hand during grasping was normalized based on the minimum and maximum angle of the hand. Then, a regression line was plotted through the normalized maximum opening angle data over the five trials for each cylinder size and test moment separately, for each individual. The slopes of these regression lines and the normalized maximum opening angles were used as separate outcome measures of proportional control.

During co-contractions to produce a switch, each of the two myosignals shows a peak. The phasing and amplitude difference of these two peaks in the myosignals during training of the TS and the NTS groups were used as outcome measures for switching control (21). Phasing was defined as the difference in time between the peak of the flexor and extensor of the wrist, respectively, where we considered a time difference between both peaks to be negatively related to skill level in switch control (21). The amplitude difference was defined as the difference in height of the peaks in the myosignal during co-contraction. The smaller the difference in peak amplitude, the higher the skill level in switch control (21). The average phasing and amplitude difference per participant was calculated for each training session. For the TS group the myosignal data of level two was used since that level was played in all training sessions. For the NTS group the three minutes of co-contraction training were used.

### Statistical analyses

Statistical analyses were performed using SPSS (IBM Corp. Released 2020. IBM SPSS Statistics for Windows, Version 27.0. Armonk, NY: IBM Corp) and R (R version 4.3.1 (2023-06-16 ucrt), packages lme4, haven, ggplot2, dplyr, broom, plotrix and mice. Significance per effect and interaction was tested by comparing the model to a “null” model where that particular effect or interaction was removed. Testing the significance of the random effect was done by comparing the mixed effects model with a linear model using only fixed effects. Missing values were filled in R using multiple imputation using the “mice” package using predictive mean matching.

#### Proportional control (effects of transfer, duration and individual differences)

In separate analyses of the Cylinder Test, the slopes of the regression lines and the averages of the normalized aperture were tested for normality with the Kolmogorov-Smirnoff test. For each outcome measure, a linear mixed model analysis was performed with Group, Test Moment and Cylinder Size as fixed effects and individual participants as random effect. Also, interaction effects were tested in the model. We chose to analyze both outcome measures in order to be able to compare the results with Van Dijk et al., 2016b.

#### Proportional and switch control (effects of transfer, duration and individual differences)

After testing for normality with the Kolmogorov-Smirnoff test, a linear mixed effects model was chosen to analyze the CRT, SHAP (key turning and page turning) and Tray Test together. Group and Test Moment were taken as fixed effects and individuals were taken as a random effect. To this end, the results of the tests were first normalized using *z*-scores. Also, interaction effects were tested in the model.

#### Myosignals of switch control during training

After testing for normality with the Kolmogorov-Smirnoff test, a linear mixed effects model was chosen to analyze the amplitude and phasing differences with a fixed effect of Group and Training Session and a random effect of individual differences.

## Results

### Descriptives

Thirty-six participants were included, 13 participants were assigned to the TS group [4 males, average age 20.8 (SD 1.3) years, 91% right handed], 12 participants to the NTS group [3 males, average age 20.0 (SD 0.9) years, 84% right handed] and 11 participants to the C group [3 males, average age 21.1 (SD 1.4) years, 93% right handed].

### Proportional control (effects of transfer, duration of training and individual differences)

**Figure 4:**
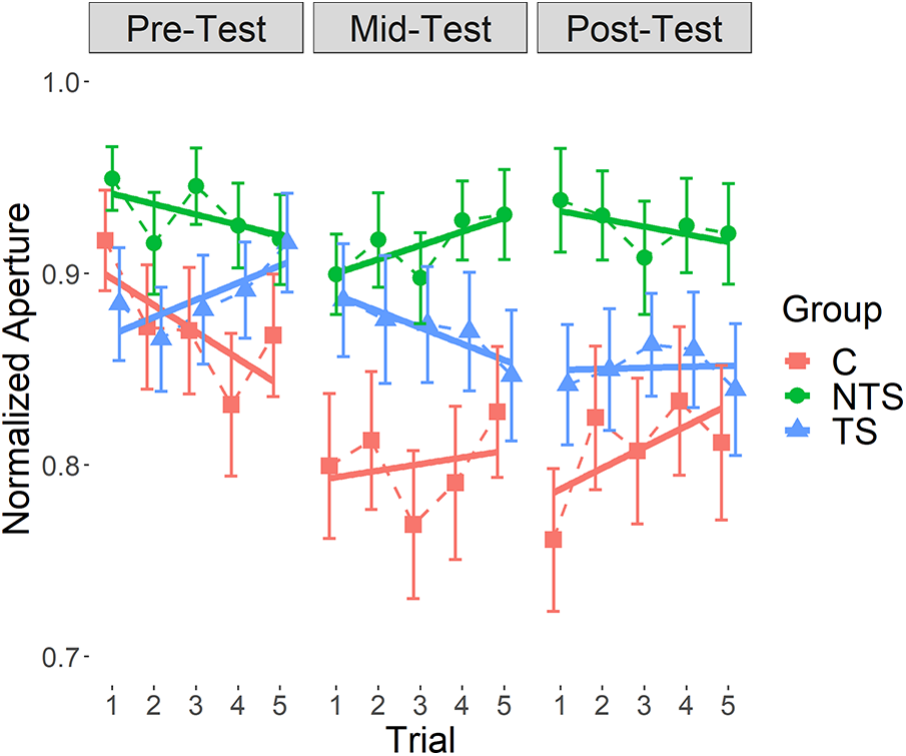
The normalized aperture collected during the cylinder test for each test moment, with standard error of the mean. Each data point is the mean across cylinder sizes per trial for each group individually connected with dashed lines, indicated with different colors and symbols. The continuous lines are the regression lines fitted through these data points, also for each group individually. The slopes of these lines are presented in Table 1. TS: Task Specific group; NTS: Non-Task Specific group; C: Control group.

The linear mixed effects model of the slopes of the regression lines from the Cylinder test showed a significant interaction between Group and Test Moment [*χ*^2^(2) = 9.21, *p* = .01]. As can be seen in Table 1, the direction of the slopes computed over the five trials was different per group for each separate test moment and differed also in direction over the test moments within each group (see Table 1 and Figure 4). We could not find a structure in the variation the directions or magnitudes of the slopes over conditions, making it difficult to interpret this interaction effect. Importantly, none of the effects of cylinder sizes were significant. No significant random effect of participants was found.

**Table 1:** Mean slope of the change in maximum aperture during pre-test, mid-test and post-test.

Group	Test	Slope^a^ (SEM)
Task-specific group	Pre-test	0.90 (0.37)
	Mid-test	−0.85 (0.58)
	Post-test	0.05 (0.61)
Non-task-specific group	Pre-test	−0.54 (0.44)
	Mid-test	0.72 (0.57)
	Post-test	−0.40 (0.52)
Control group	Pre-test	−1.39 (0.68)
	Mid-test	0.34 (0.68)
	Post-test	1.10 (0.76)

^a^ For presentation purposes, the slope (and the SEM) were multiplied by 100.

**Figure 5:**
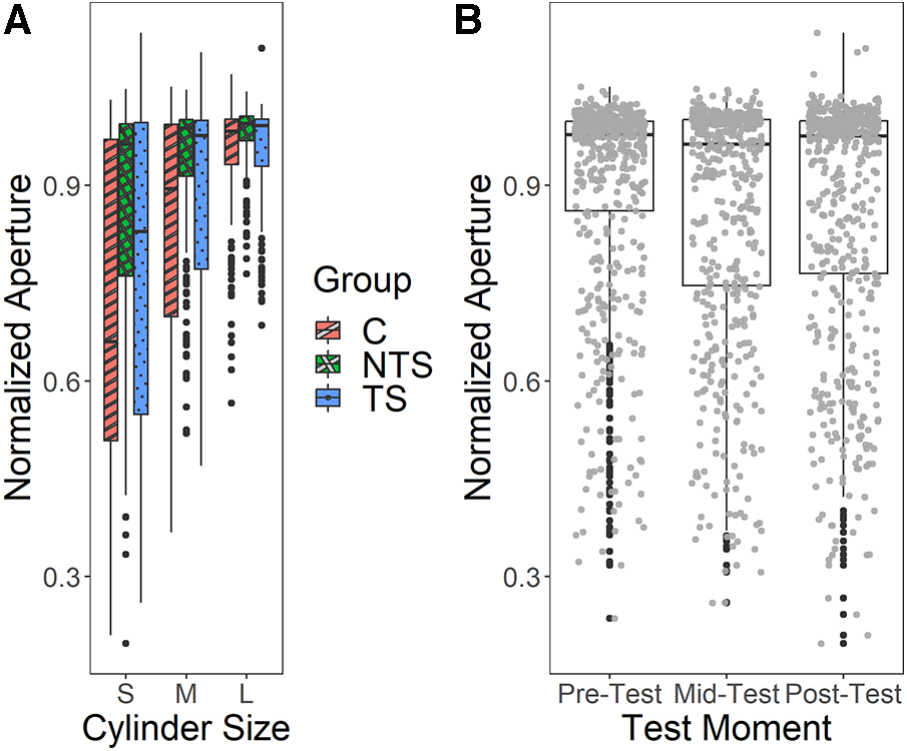
(**A**) The normalized aperture during the cylinder test for the different groups and cylinder sizes. S, small, M, medium, L, large, C, control, NTS, non-task-specific, TS, task-specific. (**B**) The normalized aperture of the Cylinder test for each test moment. Individual data points are presented in grey, outliers in black.

The linear mixed effects model of the aperture data from the Cylinder test revealed a significant interaction effect between Group and Cylinder size [*χ*^2^(4) = 58.3, *p* < .001], see Figure 5A. Figure 5A indicated a large variation in maximum aperture among participants and this variation seemed less for the NTS group compared to the TS and the C group. Furthermore, we see a trend as expected, suggesting that several participants had a smaller maximum aperture for smaller objects indicating that they scaled their maximum aperture to the object sizes. However, this scaling of maximum aperture to object size was not present in all participants, a substantial group of participants always opened the hand maximally or close to maximal, independent of the object size.

We also found a significant main effect of Test Moment [*χ*^2^(1) = 5.22, *p* = .02], shown in Figure 5B, indicating that the maximum aperture decreased over the test moments. This demonstrated that over the testing the maximum aperture was adapted to the object size with the mid-test showing overall the smallest maximum aperture. As can be seen in Figure 5B, a few data points in the post-test exceeded a 100% opening, this was due to a calibration error. Interestingly, we saw a huge individual variation which was confirmed by an overall significant main effect of individual participants [*χ*^2^(3) = 907.2, *p* < .001], indicating that participants differed significantly. As the control group did not differ from both experimental groups, transfer could not be shown.

### Proportional and switch control (effects of transfer, duration of training and individual differences)

**Figure 6:**
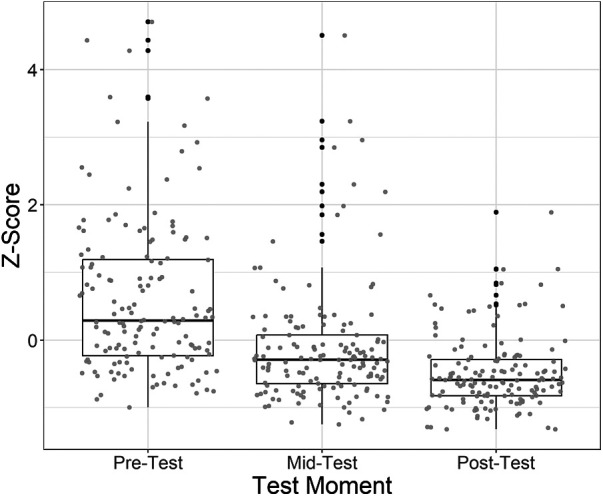
The main effect of test moment in the linear mixed effects model using the *z*-scores of the CRT, SHAP and tray test. Individual data points are represented in grey, outliers in black.

The linear mixed effects model on the data of the SHAP, CRT and Tray test showed that there was a significant fixed effect of Test Moment [*χ*^2^(2) = 48.44, *p* < .001, see Figure 6], where scores improved over test moments. Figure 6 also shows the large variation among participants, which was confirmed by the significant random effect [*χ*^2^ (6) = 36.09, *p* < .001]. None of the effects with Group were significant, implying that we could not show any transfer effect.

### Switch control during training

The data about co-contractions of the ten training sessions showed missing values (33 out of 250 total training sessions) because some participants were at times unable to produce any correct co-contraction during a training session. In total 10 participants (3 in the TS group and 7 in NTS group) produced missing values variably across all 10 sessions (Supplementary Material Table S2).

**Figure 7:**
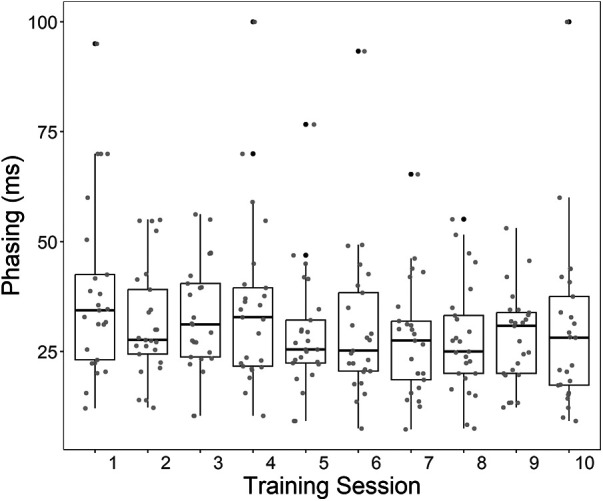
Boxplots of the phasing between the wrist flexors and extensors during co-contraction (i.e. the time difference between the peaks). Lower Phasing indicates better switch control. Individual data points are presented in grey, outliers in black.

For the amplitude differences between the two peaks of the co-contraction myosignal none of the effects were significant. For the phasing a significant effect of Training-session was found [*χ*^2^(9) = 17.23, *p* = .045], where participants improved over the ten training sessions (Figure 7). None of the effects of Group was significant. The results showed a significant effect for individual differences [*χ*^2^(1) = 66.50, *p* < .001], showing the high variation among participants.

## Discussion

The current paper aimed to establish how the training of modern bionic prosthetic hands using serious games should be organized to foster transfer from training to actual use of the prosthesis. To this end questions regarding differences in transfer among three training groups, the number of training sessions and the interaction of these factors, as well as individual differences, were addressed. These questions were asked for tasks where only proportional control applied and for tasks where both proportional and switch control were required. Overall, we found no group effects indicating that the training groups did not differ from the control group. This implies that the training did not have an effect and the differences between test moments most likely resulted from a test-effect. Since we found no training effect, the research question whether longer or shorter duration of training leads to better transfer, could not be answered. The analyses on the changes in the myosignal features in switch control training showed an increase in the alignment in of the two peaks of the co-contraction, implying that learning over training occurred for switch control. Importantly, for all the outcome measures we found large differences among individuals.

The discussion of the results below will revolve around four issues: why we did not find training effects on transfer and on duration of training, what group effects we found and what those imply for proportional control, how our results regarding learning switch control relate to the current literature and the consequences of our findings about individual differences.

### Transfer of training and duration of training

A transfer effect would imply that after a specific training, improvement in the test increases as a function of the training and not from the learning to perform tests. We did find differences between test moments in the slopes of the regression lines of the change in hand aperture in the Cylinder test, the absolute aperture and in the performance scores of the SHAP, CRT and the Tray test. However, for all these outcome measures there was no difference among the three groups on any of the three test moments. This finding implies that the control group improved as much in the tests as the two training groups, which is an indication that training did not add anything to the improvements in the test results. As such, the improvements seen over the test moments should be interpreted as a test-effect (i.e., improvement as a result of the fact that the tests have been practiced three times) and not as a result of the training. To be able to answer our research question on the duration of training, an effect of training needed to be present. However, since we found no training effect we could also not compare the differences in effect between a short and a long training duration.

Our question related to the duration of training was inspired by the work of Tabor et al. (2018) arguing that there might be beneficial effects of training of the myosignal for longer durations that usually is done in studies. They argue that these longer trainings could also lead to transfer of skill because the myocontrol skill could reach a higher level. However, in their study the transfer to actual prosthesis use was not explicitly tested. Therefore, we set out to test the interaction between the different training groups and duration of training and subsequently measure the outcomes in the functional use of the prosthesis. Importantly, our results regarding phasing in switch control during training are in agreement with the findings of Tabor et al. However, despite that we found an increase in the timing of the phasing of the two co-contraction peaks in the myosignals during training, we did not find a training effect of the two games. Hence, we concluded that the myocontrol skills in the two training games did not transfer to actual prosthesis use.

Interestingly, Van Dijk et al. found transfer results using comparable serious games as we did (9). They included four different groups; an Adaptive Catching group (comparable to our TS group), a Free catching and an Interceptive catching group (both comparable to our NTS group) and a Control group. In their study, only the Adaptive Catching group improved in prosthesis control after training which supported their claim that transfer was found due to the task specificity introduced in the game the Adaptive Catching group utilized. Therefore, the results of Van Dijk et al. were different than ours in that they found a transfer effect for the groups training the task-specific task. One explanation for this finding could be that the serious game used in the current study was slightly altered compared to theirs. In our version participants could only catch the falling block after a correct co-contraction was made, a requirement that turned out to be difficult for most participants especially in the first training sessions. This requirement was not incorporated in the game used by Van Dijk et al. which could have allowed their participants more practice with the falling block than participants in our study, most probably resulting in more task-specific training trials and perhaps therefor transfer. It might be that the game needs to be designed in such a way that performance in one type of training (proportional control) does not depend on the other performance (switching control) to ensure that both types of training are done.

### Group effects

Interestingly, a few group effects were found. The Group and Test moment interaction on the slopes of the regression lines in the Cylinder test was found to be statistically significant. However, no systematicity in the interaction could be revealed and further interpretation is not possible. On the absolute aperture we found an interaction effect between Group and Cylinder size. It was surprising to see that the C-group performed quite similar as the TS group. Although the C-group trained playing both serious games and were exposed to the grasping task, the grabber was controlled with a computer mouse and keyboard presses. This implies that although the C-group did not train myocontrol, they received a lot of visual feedback (i.e., the same sparks presented in the TS group) on how their hand opening scaled to the object size as the TS group did. Furthermore, participants in the C-group were able to start catching the blocks in the task-specific serious game much quicker than participants in the TS-group. This might be explained by the fact that the C-group did not have to make a correct co-contraction first, which was a difficult requirement for the TS-group. The C-group was exposed to the block catching mechanics consistently from the start. It might be that this feedback in training helped the C-group to scale the hand aperture of the prosthesis simulator in the test sessions.

With such a finding the question should be asked if this result has any clinical applications. For example, can participants train with a joystick and a version of this game to set up a perception-action coupling based on the primary information provided by the game? And might that training transfer to actual prosthesis control? It could be the case that primary information of a serious game can be picked up regardless if it is controlled by myosignals or a computer mouse and keyboard. This might be partially supported by another study where transfer is thought of as calibration of an existing perception-action coupling based on the information presented during training (46). It might be possible that playing the serious game trains the function that is being performed (grasping an object) and that this function is more primary than the movements with which this function is controlled. Whether this phenomenon could play a role in transfer from serious game training to actual prosthesis control cannot be answered with our results and needs to be investigated in the future.

### Switch control

Like Tabor et al. we found that over sessions a general improvement in phasing occurred, although in our study the decrease in the time difference of the two myosignal peaks occurred in the first training sessions while participants in Tabor et al. mostly improved in the later sessions (see Figure 7). Note that also Heerschop et al. (2022) found that participants improved in their phasing mostly in the first five sessions after training with a serious game. As to why phasing improved but amplitude did not could partly be related to how the system detected correct co-contractions. For a correct co-contraction both peaks of the myosignal needed to be 40% of maximum voluntary contraction and within 350 ms of each other. These set of restrictions allow for much more variability in amplitude difference than in phasing because a change in amplitude difference does not have a direct effect on whether a co-contraction is correct or not, and the phasing does. In other words, participants can vary wildly in amplitude difference as long as both peaks are above 40% while varying in phasing can only be done up to a difference of 350. Therefore the nature of the control might push the neuromotor system to improve their phasing while amplitude differences are less restricted.

### Individual differences

We found individual differences in almost all analyses using a linear mixed effects model, indicating a substantial amount of variation in the data that did not come from the experimental conditions. The fact that individuals can differ in both their initial conditions and in their improvement is not a new finding (e.g. (28, 40, 54, 55), however when developing training programs for upper limb prosthesis use it might deserve more attention. A first step might be to switch from analyses on mean behavior to appreciating individual difference. Although this entails methodological challenges that need to be overcome (cf. Anderson & Williams, 2022), the current findings indicate that this is the route to go. Such individual analyses might help in creating profiles or categories of participants based on their individual learning process. A next step could then be that a specific motor learning training could be created specifically tailored to each profile. Then individual differences would not be an additional factor of variability but a phenomenon that could be exploited to enhance learning for every type of motor learner. Anecdotally it was found in the current study that some participants could quickly learn to play the game while others had troubles with learning to play the game throughout the training sessions. What determined this difference would be an interesting topic for further research.

The inclusion of serious games in this process could be exceptionally beneficial, since the type of game can be tailored to the specific profile of a user. Note that perhaps a game can be used as a screening tool to distinguish different motor learning profiles early in the rehabilitation process. Moreover, a game can be designed to provide varied feedback or challenges to different performances, for instance, difficulty levels or type of feedback can be individualized. Including such a serious game would ensure that everyone would receive the type training best suited for them, without the need for therapists to create different training schedules per person.

### Limitations

A few limitations in the current study can be identified. First, motivation during training could have been affecting the participants' performance. They were asked to train several sessions with the serious games. Based on informal conversations with participants it was found that the serious games in their current form were limited in their motivation to progress. In many cases, participants got demotivated over time and found themselves distracted while playing the games in the later training sessions. This could have influenced their performance in a negative way. Future studies should design serious games that have more to offer, such as different types of feedback, levels, competition systems or a leaderboard. This could increase motivation, engagement and hopefully this could lead to transfer to actual prosthesis use after training with a serious game. Second, the eligibility criteria could have affected the generalizability of our study. All participants were able bodied students recruited from the university, while a large part of the population of prosthesis users is much older. Able-bodied persons are not entirely comparable to prosthesis users, although research has shown that there are similarities in myosignals between able-bodied persons and prosthesis users (56). However, the difference in age could be a factor in the effectiveness of using a serious game as a training tool for prosthesis control, something that needs to be investigated further. Therefore, future studies should also include actual prosthesis users. Furthermore, a suggestion for real world applicability of further research would be to study the ratio of successful and unsuccessful co-contractions. In addition, it would be interesting to explore whether providing participants with the option to adjust the co-contraction requirements to their personal preferences would result in improved outcomes.

## Conclusions

We found that for proportional control there were differences in improvement between training groups. However, there was no structure found in these differences so we were unable to say which training group improved more than both other games. For tasks where proportional and switch control were needed and for only proportional control tasks we found that participants of all groups improved over the testing sessions. This indicated that not a transfer effect but a testing effect was found in the current study. For the learning of switch control we also found no difference between groups even though an overall improvement was observed. An important finding across all analyses was that significant individual differences were found throughout our study which not just means that motor learning is different for each person but that these individual differences should be taken into account in future studies in prosthesis use and in its translation to rehabilitation practice.

## Data Availability

The raw data supporting the conclusions of this article will be made available by the authors, without undue reservation. To receive the raw data, please contact the corresponding author.
