# Corrigendum: Assessing effectiveness of serious game training designed to assist in upper limb prosthesis rehabilitation

**DOI:** 10.3389/fresc.2024.1532227

**Published:** 2024-12-09

**Authors:** Bart Maas, Corry K. Van Der Sluis, Raoul M. Bongers

**Affiliations:** ^1^ Department of Human Movement Sciences, University Medical Center Groningen, University of Groningen, Groningen, Netherlands; ^2^ Department of Rehabilitation Medicine, University Medical Center Groningen, University of Groningen, Groningen, Netherlands

**Keywords:** prosthesis, rehabilitation, serious games, upper limb prosthesis, task specificity

A Corrigendum on Assessing effectiveness of serious game training designed to assist in upper limb prothesis rehabilitation By Maas B, Van Der Sluis CK, Bongers RM. (2024). Front. Rehabil. Sci. 5:1353077. doi: 10.3389/fresc.2024.1353077

**Error in the Article Title**

In the published article, there was an error in the article title. Instead of “Assessing effectiveness of serious game training designed to assist in upper limb prothesis rehabilitation”, it should be “Assessing effectiveness of serious game training designed to assist in upper limb prosthesis rehabilitation”.

The authors apologize for this error and state that this does not change the scientific conclusions of the article in any way. The original article has been updated.

