# Supplementary material for: Assessing effectiveness of serious game training designed to assist in upper limb prosthesis rehabilitation
Source: Front Rehabil Sci. 2024 Jan 29;5:1353077. doi: 10.3389/fresc.2024.1353077 (PMC10859406; doi:10.3389/fresc.2024.1353077)
Supplement: Supplementary file 1 [file Table2.docx]

| Table 2. Missing values for the switch control analysis. | | |
| --- | --- | --- |
| Training session | TS (n = 13), participant number | NTS (n = 12), participant number |
| 1 | 11 | 1, 2, 4, 12 |
| 2 | - | 1, 2, 5 |
| 3 | - | 1, 2, 9 |
| 4 | - | 1, 2, 3 |
| 5 | 11 | 3, 9 |
| 6 | 4 | 1, 2 |
| 7 | 4 | 3 |
| 8 | 2 | 3, 8 |
| 9 | 2, 4 | 2, 3 |
| 10 | 2 | 1, 4, 9 |
